# Differential item functioning (DIF) of SF-12 and Q-LES-Q-SF items among french substance users

**DOI:** 10.1186/s12955-015-0365-7

**Published:** 2015-10-24

**Authors:** Stéphanie Bourion-Bédès, Raymund Schwan, Vincent Laprevote, Alex Bédès, Jean-Louis Bonnet, Cédric Baumann

**Affiliations:** Service Médico Psychologique Régional, 57 073 Metz, France; CSAPA (Health Care Centre of Accompaniment and Prevention in Addictology), University Hospital of Nancy, Nancy, France; EA4360 APEMAC, University of Lorraine, Nancy, France; ANPAA 15- CSAPA (Health Care Centre of Accompaniment and Prevention in Addictology), Saint-Flour, Cantal, France; ANPAA 15- CSAPA (Health Care Centre of Accompaniment and Prevention in Addictology), Aurillac, Cantal, France; ESPRI-BioBase Unit, PARC, University Hospital of Nancy, Nancy, France

**Keywords:** Q-LES-Q-SF, Differential Item Functioning, Self-reported health status, Alcohol dependence, Opiate dependence

## Abstract

**Background:**

Differential Item Functioning (DIF) is investigated to ensure that each item displays a consistent pattern of responses irrespective of the characteristics of the respondents. Assessing DIF helps to understand the nature of instruments, to assess the quality of a measure and to interpret results. This study aimed to examine whether the items of the Quality of Life Enjoyment and Satisfaction Questionnaire-Short Form (Q-LES-Q-SF) and Short-Form 12 (SF-12) exhibit DIF.

**Method:**

A total of 124 outpatients diagnosed with substance dependence participated in a cross-sectional, multicenter study. In addition to the Q-LES-Q-SF and SF-12 results, demographic data such as age, sex, type of substance dependence and education level were collected. Rasch analysis was conducted (using RUMM2020 software) to assess DIF of the Q-LES-Q-SF and SF-12 items.

**Results:**

For SF-12, significant age-related uniform DIF was found in two of the 12 items, and sex-related DIF was found in one of the 12 items. All of the observed DIF effects in SF-12 were found among the mental health items. Three items showed DIF on the Q-LES-Q-SF; however, the impact of DIF item on the delta score calculation for the comparisons of self-reported health status between the groups was minimal in the SF-12 and small in the Q-LES-Q-SF.

**Conclusion:**

These results indicated that no major measurement bias affects the validity of the self-reported health status assessed using the Q-LES-Q-SF or SF-12. Thus, these questionnaires are largely robust measures of self-reported health status among substance users.

## Background

Interest in patient-reported outcomes in addiction research has grown rapidly over the last few decades given the chronic, relapsing nature of drug use and the negative consequences of drug use on various life domains [1, 2]. Measurement of self-reported health status has become an important clinical and research tool for assessing the health of patients with substance use disorders [3]. Generic and specific instruments are applied to measure the self-reported health status of substance users; however, due to the subjective nature of health status self-reports, individuals from various groups may interpret the wording of the items in manners that are extraneous to the assessment, which means that items may be functioning differently [4]. Much of the validity assessment of self-reported health status instruments has focused on factor structure. One aspect of validity assessment that is clearly lacking in the literature is the assessment of invariance. Measurement invariance is a psychometric property of a scale that measures a latent construct across different respondent groups. Establishing the invariance of self-reported health status instrument items is essential for between-group comparisons and for further understanding psychological phenomena [4]. A lack of invariance can question the validity of an instrument because a key assumption in measurement is that characteristics of the respondents that are unrelated to the construct being measured (e.g., country, language or culture of the respondents) do not affect their responses to the items [5, 6]. The differential item functioning (DIF) is a form of violation of measurement invariance, in other words, DIF represents a situation where the measurement invariance does not hold [7]. Ideally, the pattern of responses should be invariant across groups who are at the same level on the latent variable, that means they have the same probability of responding to the question, whatever theirs characteristics (young vs old people, males vs females, etc.) [8, 9]. If DIF is present, then the observed group differences at least partially reflect something other than the latent variable, such as different interpretations of the item between different groups. DIF can result in biased between-group comparisons because the response patterns may reflect attributes other than that which the instrument is intended to measure [10,11].

The Quality of Life Enjoyment and Satisfaction Questionnaire-Short Form (Q-LES-Q-SF) is a self-report measure designed to assess the degree of enjoyment and satisfaction in daily functioning. It has been shown to be a reliable and valid unidimensional instrument in several languages and in different populations with psychiatric illnesses, including adults with ADHD, generalized anxiety disorder, bipolar disorder or substance dependence [12–14]. Although many studies have demonstrated the satisfactory psychometric properties of the Q-LES-Q-SF, DIF assessment of Q-LES-Q-SF items to determine their invariance has never been demonstrated across sociodemographic and clinical groups. Prior studies examined DIF for the Short-Form 36 (SF-36) and Short-Form 12 (SF-12), two generic measures of health. One study examining DIF with respect to demographic groups in the SF-12 items in a national sample of the USA found significant age-related DIF in eight of the 12 items, sex-related DIF for four of the 12 items, education-related DIF for six of the 12 items, and ethnic-related DIF for three of the 12 items. [10]. Several methods have been applied to assess the invariance of items, particularly the DIF, in health-related scales: structural equation modeling, ordinal logistic regression, and Rasch analysis using item response theory (IRT) analysis and contingency tables [15]. Given the widespread use of self-reported health status instruments in both clinical and research samples of substance users and the recommended use of generic and disease-/population-specific instruments, this study aimed to investigate the DIF of Q-LES-Q-SF and SF-12 items in a French sample of substance users across groups classified according to sex, age, education level and type of substance dependence.

## Methods

### Data source and sampling

The data were collected from a French cross-sectional, multicenter study. The outpatients who met the DSM-IV criteria for alcohol or opiate dependence were sampled from four French specialized addiction treatment centers in two regions of France [16]. The patients were assigned to the alcoholic or the opiate group according to their main dependence (alcohol or opiate) on axis I of the DSM-IV. The diagnosis was made by clinicians certified in addiction pathologies who were familiar with the DSM-IV. The study protocol was approved by the Institutional Review Board (Comité National Informatique et Liberté DR-2013-156), ensuring the confidentiality of the patient information.

### The Q-LES-Q-SF questionnaire

The Q-LES-Q-SF is a self-report instrument comprising 16 items derived from the general activities scale of the original 93-item form [17]. It consists of fourteen items assessing satisfaction with his/her physical health, social relations, ability to function in daily life, physical mobility, mood, family relations, sexual drive and interest, ability to perform hobbies, work, leisure activities, and household activities, economic status, living/housing situation, vision and overall well-being. Each of the 14 items is rated on a 5-point scale that indicates the degree of enjoyment or satisfaction experienced during the past week. The total score of all 14 items is computed (ranging from 14 to 70) and is expressed as a percentage (1–100) of the maximum total score. Higher scores on the Q-LES-Q-SF indicate greater contentment or satisfaction. The instrument also includes two additional items measuring satisfaction with medication and overall life satisfaction that are not included in the overall score. As the French version of the Q-LES-Q-SF yielded valid and reliable clinical assessments of self-reported health status, it was used in this study [14].

### The SF-12 questionnaire

The SF-12 is a well-known generic self-report health status instrument that includes a subset of 12 items from SF-36 [18]. Information from all 12 items is used to calculate a physical component score (PCS) representing the physical health (PH) domain and a mental component score (MCS) representing the mental health (MH) domain. All of the scores are transformed to a standardized 0–100 score. Higher scores indicate a better self-reported health status.

### Other data

The sociodemographic data collected included age, sex and education level. Age (years) was dichotomized using the median cutoff value. The main substance dependence was determined by a trained clinician, who completed a questionnaire used in routine clinical care. The patients were assigned to the alcoholic or the opiate group according their main dependence.

### Statistics

As a first step, continuous variables were expressed as the means (standard deviation) or medians as appropriate for continuous variables, and categorical variables were expressed as numbers or percentages.

#### Confirmatory factor analysis

The structural validity of the Q-LES-Q-SF questionnaire was investigated via confirmatory factor analysis (CFA) using categorical factor indicators and a robust weighted least squares estimator. Analysis was performed using Mplus 6.12 (Muthe´n & Muthe´n, Los Angeles, CA, USA). The model was judged as good if the root mean square error of approximation (RMSEA) < 0.08, the comparative fit index (CFI) > 0.9, and the Tucker–Lewis index (TLI) > 0.9 or as excellent if RMSEA < 0.05, CFI > 0.95, and TLI > 0.95 [19,20]. The factor structure of SF-12 results among people with mental disorders is well known [21].

#### Rasch analysis

Rasch analysis (a member of the family of item response theory models) relates latent trait(s) of interest to the probability of responses to items on the assessment. It is a model‐based measurement in which latent trait level estimates depend on both persons’ responses and on the properties of the items. Rasch analysis models the latent variable as a logistic function of observed item responses, named Item Characteristic Curve (ICC). It is a curve that represents the relationship between the probability of “correct” response (where “correct” can be defined by the subject's expected item-level response to an item coherently with the latent trait) and the latent trait (self-reported health status) [22, 23]. Rasch analysis tests whether the data fit the model by assessing whether the response pattern observed in the data corresponds to the theoretical pattern expected by the model [24]. The Rasch model provides a way of relating item difficulty to respondent characteristics. Rasch analysis is described in detail elsewhere [24, 25]. The item fit was explored based on standardized residuals (item and person-fit residuals expected to range between ± 2.5 units) and examination of the ICCs. The internal consistency of the domains was examined using a person separation index (PSI). PSI values of 0.90 or greater indicated excellent results [26].

The RUMM software program was employed to assess DIF via Rasch analysis. Analysis of variance (ANOVA) of the standardized response residuals for each item was conducted across each level of the factors and the class interval (i.e., at different levels of the trait) [27, 28]. Analysis was conducted using both statistical and ad hoc graphical procedures to illustrate DIF. DIF was illustrated using the ICCs, which show the expected item score as a function of the underlying construct (e.g., physical functioning ability for the physical health (PH) dimension). The location parameter (theta, θ) reflects the position of the item along the continuum (in logits). Two types of DIF may be identified: uniform and non-uniform DIF. Uniform DIF indicates a consistent systematic difference in the responses to an item between the groups across the entire range of the attribute being measured (e.g., the bias is constant for all values of the latent variables). Graphically, the curves are displaced by a shift in their location on the theta continuum of variation, and uniform DIF is reflected in the ICC by parallel lines, showing a constant difference between the groups. Non-uniform DIF indicates varying difference across levels of the attribute, appearing as non-parallel lines in the ICC [7]. Every item of SF-12 and the Q-LES-Q-SF was examined for DIF across four parameters within the sample: sex (male and female), age (above and below the median), education level (junior and senior high school) and type of substance dependence (alcohol and opiate dependence). The analysis was conducted for the 2 SF-12 dimensions (mental health (MH) and physical health (PH)) and for the Q-LES-Q-SF, once its unidimensionality was established.

#### Comparative analysis

To describe impact of the DIF of each item in both SF-12 and Q-LES-Q-SF on its corresponding score, a new score excluding the item displaying DIF, was calculated and transformed to a 0–100 standardized score. The difference in the scores on both the SF-12 and Q-LES-Q-SF questionnaires between the inclusion and exclusion of DIF (∆ Score) was compared between the modalities of each variable-related DIF using a paired-*t*-test.

Rasch analysis was conducted using RUMM2020 software (Rumm Laboratory, Perth, Western Australia), and descriptive and comparative analysis was performed using SAS v9.3 (SAS Inc., Cary, NC, USA). The overall significance level was set to 0.05.

## Results

### Description of the sample

Overall, 124 patients were included in the study. Most of the patients were male (83.3 %). The mean age was 39.2 years (11.7), and the median age was 36 years. According to the DSM-IV criteria, 57 (46 %) patients suffered from alcohol dependence, and 67 patients (54 %) from opiate dependence. The majority of the sample (72 %) reported a low level of education (junior high school). The Q-LES-Q-SF score was 56.9 (SD = 20). The SF-12 scores were 58.9 (SD = 22.9) and 49.5 (SD = 22.3) for the PCS and the MCS, respectively.

### Unidimensionality, item fit and internal consistency

For the Q-LES-Q-SF, the CFA confirmed a one factor model in which RMSEA = 0.077 (90%CI [0.054 - 0.098]), CFI = 0.968 and TLI = 0.962, with loadings between 0.523 and 0.851. For the Q-LES-Q-SF, the PSI was 0.90, and the item residuals were between −2.5 and +2.5 with no statistical significance. The PH and MH domains of SF-12 demonstrated PSIs of 0.92 and 0.93, respectively, indicating good internal consistency.

### Differential Item Functioning

#### DIF for the Q-LES-Q-SF

**Fig. 1:**
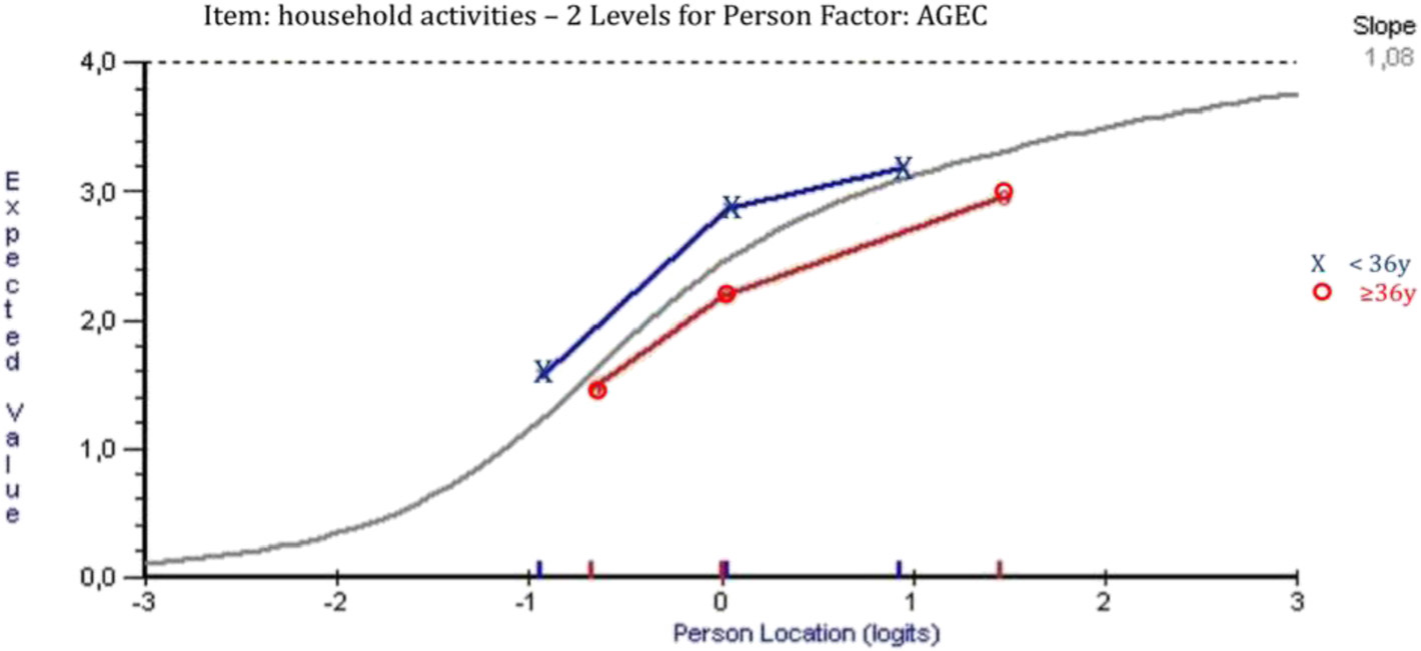
Differential item functioning graph of the age group for item 4 from the Q-LES-Q-SF

**Fig. 2:**
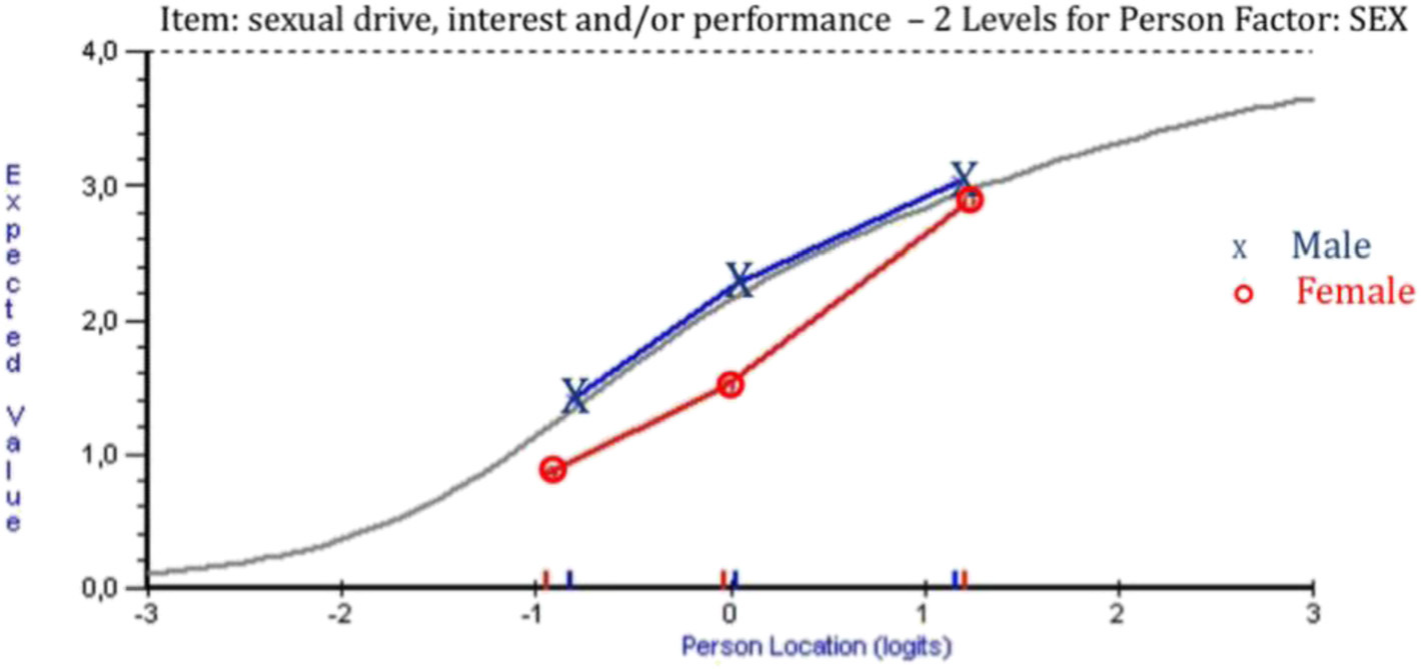
Differential item functioning graphs of the sex group for item 9 from the Q-LES-Q-SF

**Fig. 3:**
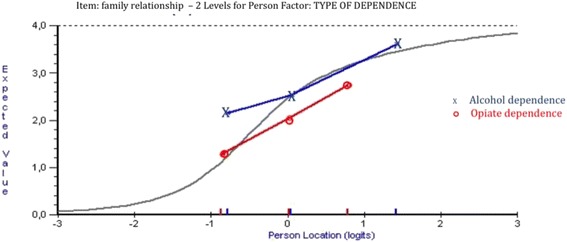
Differential item functioning graphs of the type of substance dependence group for item 6 from the Q-LES-Q-SF

Three of the items in the Q-LES-Q-SF showed uniform DIF: item 4 (“*household activities*”) for the age group, item 6 (*“family relationships*”) for the substance dependence type group and item 9 (“*sexual drive, interest and/or performance*”) for the sex group (Table 1). For the age group, item 4 (*“household activities”)* showed statistically significant and graphically remarkable uniform DIF (Fig. 1). The younger participants were more likely to report that they had no difficulty with household activities than the older participants, despite equivalent level of characteristics for the latent trait. For item 9 (“*sexual drive, interest and/or performance”*), men were more likely to report a high item-score (high probability of success of the item 9) than women; therefore, the item difficulty was higher for women. This result is presented in Fig. 2. For item 6 (*“family relationship”*), patients with alcohol dependence were more likely to report that they were satisfied with family relationships than patients with opiate dependence (Fig. 3).

**Table 1:** Uniform Differential Item Functioning (DIF) in the Q-LES-Q-SF items according to age, education level, sex and type of substance dependence

	Age		Education level		Sex		Type of substance dependence	
	MS total DIF	*P*	MS total DIF	*P*	MS total DIF	*P*	MS total DIF	*P*
Items from the Q-LES-Q-SF								
1. Physical health	0.003	0.948	0.044	0.795	0.264	0.524	1.370	0.154
2. Mood	0.047	0.811	0.142	0.686	0.016	0.891	0.437	0.467
3. Work	0.328	0.599	0.012	0.922	3.662	0.075	2.473	0.140
4. Household activities	8.782	0.008^a^	0.774	0.425	0.088	0.793	1.360	0.299
5. Social relationships	0.020	0.877	1.264	0.208	0.004	0.948	1.543	0.165
6. Family relationships	2.366	0.136	0.090	0.775	0.251	0.628	11.12	0.001^a^
7. Leisure time activities	0.279	0.591	0.004	0.952	0.391	0.537	0.424	0.518
8. Ability to function in daily life	0.505	0.523	1.403	0.288	0.031	0.872	0.917	0.381
9. Sexual drive, interest and/or performance	0.172	0.710	0.119	0.762	4.975	0.044^a^	1.381	0.290
10. Economic status	3.203	0.099	0.949	0.349	0.460	0.530	1.544	0.242
11. Living/ housing situation	2.072	0.165	0.005	0.949	1.010	0.379	1.677	0.245
12. Ability to get around physically without feeling dizzy or falling	0.145	0.681	0.317	0.560	0.002	0.961	1.410	0.212
13. Vision in terms of ability to do work or hobbies	1.318	0.301	0.022	0.894	0.198	0.690	2.108	0.192
14. Overall sense of well-being	0.080	0.725	0.001	0.99	0.050	0.777	0.133	0.653

MS: mean square

^a^Uniform DIF: total DIF p < 0.05

The difference in the scores on the Q-LES-Q-SF questionnaire between including and excluding the items displaying DIF (∆ Score) are presented in Table 2. Removing items 4 and 6 impacted the results of the self-reported heath status. There was a statistically significant delta score according to age for item 4 and according to the type of substance dependence for item 6. In the age < 36 years group, the mean score including all 14 items was higher than the mean score excluding item 4 (∆ Score = +0.65), whereas in the age ≥ 36 years group, the score including all items was lower than the mean score excluding item 4 (∆ Score = −0.29). This difference between the ∆ Scores was significant (*p* = 0.009). Similarly, excluding item 6 led to difference in the reported scores between the two groups of substance users. In the alcohol dependence group, excluding item 6 was accompanied by an overestimation of the score (∆ Score = +0.87), whereas the opposite relationship was observed in the opiate dependence group (∆ Score = −0.32). The difference between the two ∆ Scores was statistically significant (*p* = 0.001).

**Table 2:** Comparison of the difference in the Q-LES-Q-SF scores between including and excluding the items displaying DIF between the groups of each variable-related DIF

Variable-related DIF	∆ Score Q-LES-Q-SF^a^ Mean (SD)	*P value*
Age (item 4)		0.009
Age < 36 years (*n* =72)	0.65 (1.5)	
Age ≥ 36 years (*n*=52)	-0.29 (2.3)	
Type of substance dependence (item 6)		0.001
Alcohol dependence (*n*=57)	0.87 (2.0)	
Opiate dependence (*n*=67)	-0.32 (2.1)	
Sex (item 9)		0.06
Male (*n*=103)	0.01 (1.8)	
Female (*n*=21)	-0.9 (1.8)	

^a^∆ Score Q-LES-Q-SF = (score including all 14 items) – (score excluding the item displaying DIF)

(Scores were on a 0–100 standardized scale)

#### DIF for SF-12

No items in the PH dimension showed any DIF across the four groups (Table 3). Three MH items showed statistically and graphically significant uniform DIF. For sex, the MH1 item *“Accomplished less (emotional)”* showed statistically significant and graphically remarkable uniform DIF. At the same mental health location, men were more likely to report a high-item score than women (*p* = 0.001); therefore, the item difficulty was higher for women. Moderate DIFs were shown for the MH2 item *“worked less carefully”* and the MH4 item *“felt downhearted”* according to age. The younger participants were more likely to report a high-item score than the older participants. No DIF was detected according to the type of substance dependence.

**Table 3:** Uniform Differential Item Functioning (DIF) in the SF-12 items according to age, education level, sex and type of substance dependence

	Age		Education level		Sex		Type of substance dependence	
	MS total DIF	*P*	MS total DIF	*P*	MS total DIF	*P*	MS total DIF	*P*
SF-12 Physical Health domain								
PH1. General health	0.749	0.357	0.180	0.649	0.034	0.845	2.359	0.099
PH2. Moderate activities	1.312	0.165	0.004	0.939	0.841	0.270	0.098	0.703
PH3. Climbing stairs	1.249	0.193	0.015	0.887	0.310	0.503	0.664	0.334
PH4. Accomplished less (physical)	0.221	0.596	1.812	0.126	0.777	0.320	0.294	0.540
PH5. Limited in work	0.007	0.910	0.130	0.623	0.502	0.317	0.171	0.566
PH6. Pain	0.532	0.407	0.006	0.928	0.052	0.796	1.299	0.196
SF-12 Mental Health domain								
MH1: Accomplished less (emotional)	0.136	0.635	0.473	0.394	7.147	0.001^a^	0.050	0.784
MH2: Worked less carefully	2.867	0.042^a^	0.910	0.250	0.765	0.299	0.193	0.602
MH3: Felt calm	0.484	0.466	0.105	0.737	0.001	0.981	0.179	0.651
MH4: Felt downhearted	3.113	0.043^a^	0.006	0.923	0.066	0.766	2.534	0.064
MH5: Had energy	0.442	0.560	0.016	0.911	4.036	0.054	2.116	0.197
MH6: Social activities	3.002	0.079	0.053	0.813	0.354	0.539	0.007	0.933

MS: mean square

^a^Uniform DIF: total DIF p < 0.05

The comparisons of differences in MH scores including and excluding the items displaying DIF (∆ MH score of SF-12) were not significant according to sex (item MH1) or age (items MH2 and MH4); therefore, we did not detect any significant bias related to DIF for the MH domain (Table 4).

**Table 4:** Comparison of the difference in the SF-12 scores between including and excluding the items displaying DIF between the groups of each variable-related DIF

Variable-related DIF	∆ Score SF-12^a^	*P value*
	Mean (SD)	
Sex (item MH1)		0.98
Male (*n* =103)	0.05 (3.0)	
Female (*n*=21)	0.03 (3.1)	
Age (item MH2)		0.73
Age < 36 years (*n* =72)	-0.64 (2.9)	
Age ≥ 36 years (*n*=52)	-0.45 (3.1	
Age (item MH4)		0.93
Age < 36 years (*n* =72)	0.96 (9.4)	
Age ≥ 36 years (*n*=52)	1.12 (9.9)	

^a^∆ Score SF-12 = (score including all 12 items) – (score excluding the item displaying DIF)

(Scores were on a 0–100 standardized scale)

## Discussion

This study examined whether the response pattern to certain items of the Q-LES-Q-SF and SF-12 varied in a sample of substance users. Once the dimensionality of the instruments was established, significant age-related DIF and sex-related DIF were identified for only two items and one item, respectively, in the MH dimension of SF-12. Three items showed uniform DIF on the Q-LES-Q-SF. In contrast to the result that the older group was associated with higher scores on downhearted items in prior studies of DIF in the items of SF-12 or SF-36 across demographic characteristics [10, 29], the results of this study showed that the younger participants were more likely to report a high item-score than the older participants for the items of SF-12 such as *“worked less carefully”* and *“felt downhearted”*. Consistent with prior studies that reported evidence of sex-related DIF, this study revealed a sex-related DIF for the item *“Accomplished less (emotional*)”, implying that men were more likely to report a high item-score than women. As proposed by Fleishman et al., one clinical interpretation for items demonstrating sex-related DIF may be that men are more likely to adopt a stoic perspective and refrain from providing responses that imply weakness [29]. As prior health status studies have found that there are differences in self-reported health status according to age, sex and education level, it was important to investigate DIF for all 14 items in the Q-LES-Q-SF. As expected, younger people reported greater satisfaction with their ability to perform household activities than older people. For the items *“sexual drive, interest and/or performance”* and *“family relationship”*, clinical interpretations are available. Sex differences may arise for the item *“sexual drive, interest and/or performance*” because men are more likely to take a stoic orientation and respond favorably to this item, as an alternative response implies weakness. For the item *“family relationship”*, a substance dependence difference might arise because substance use may be more likely to be construed as a coping mechanism among those in the close family circle according to individuals with alcohol dependence than according to individuals with opiate dependence.

Several options are available for addressing DIF. In the literature, six methods for ameliorating DIF in existing measures are used: (1) construct separate measures, (2) reword items to minimize bias, (3) select other items that are more universally applicable, (4) remove the biased items from the total score, (5) adjust the scores by transformation or (6) reweight the biased items [10, 30]. Excluding the items displaying DIF in SF-12, the results of the between-group comparisons were similar to those of the original between-group comparisons. For the Q-LES-Q-SF, two outcomes of the statistical analysis were altered by excluding the items displaying DIF. Although these differences were significant, the impact of DIF on the delta score calculation between the groups remained clinically negligible (∆ score of the self-reported health status < 1 point) compared with the minimal difference of 5 points usually deemed relevant in the literature [31, 32]. Because of the unclear comprehension of the practical meaning of a significant DIF, it is difficult to interpret the difference in DIF impact between the two questionnaires used. The items of the Q-LES-Q-SF might be more sensitive than those formulated in the generic SF-12. Nevertheless, no scientific rational supports this hypothesis. Removing an item with DIF from a scale is not without consequences on its psychometric properties; however, if various characteristics (e.g., sex, age, and so on) are affected by a DIF phenomenon within a scale, then it is difficult to adjust for all sources of DIF in a multivariate analysis [33]. Recent findings based on simulated datasets suggest that the percentage of items in a scale affected by a uniform DIF should be taken into account. If less than 50 % of the scale items are affected by a uniform DIF with a small effect size, then the resulting measurement bias at the scale level would not be meaningful, regardless of the level of difficulty of these DIF [34].

Although the interesting findings of this study support the use of the Q-LES-Q-SF and the generic SF-12 to evaluate health status among substance users, some limitations of this study remain. First, all of the patients were recruited through specialty treatment services; therefore, the sample cannot be considered as a reflection of patients with alcohol or opiate dependence in routine medical practice. Second, the groups were defined according to a median threshold, and it is possible that other thresholds may have produced different results.

## Conclusion

The results of this study have both practical and theoretical implications. From a practical perspective, few items displayed DIF. The results indicated that no major measurement bias affects the validity of the quality of life findings as assessed by the Q-LES-Q-SF and SF-12, which are largely robust measures of self-reported health status among substance users. From a theoretical perspective, a further understanding of how sociodemographic characteristics may influence the manner in which substance users interpret and respond to questions assessing self-reported health status is needed.

The results of this study support the regular performance of DIF determination as a standard measurement of validity assessment for self-report health status measures and suggest the concurrent evaluation of specific and widely used generic instruments among substance users.
